# Maximum Sum of Evidence—An Evidence-Based Solution to Object Pose Estimation in Point Cloud Data

**DOI:** 10.3390/s21196473

**Published:** 2021-09-28

**Authors:** Tyson Phillips, Tim D’Adamo, Peter McAree

**Affiliations:** School of Mechanical and Mining Engineering, The University of Queensland, Brisbane, QLD 4072, Australia; t.dadamo@uq.edu.au (T.D.); p.mcaree@uq.edu.au (P.M.)

**Keywords:** pose estimation, object classification, localisation, perception, LiDAR

## Abstract

The capability to estimate the pose of known geometry from point cloud data is a frequently arising requirement in robotics and automation applications. This problem is directly addressed by Iterative Closest Point (ICP), however, this method has several limitations and lacks robustness. This paper makes the case for an alternative method that seeks to find the most likely solution based on available evidence. Specifically, an evidence-based metric is described that seeks to find the pose of the object that would maximise the conditional likelihood of reproducing the observed range measurements. A seedless search heuristic is also provided to find the most likely pose estimate in light of these measurements. The method is demonstrated to provide for pose estimation (2D and 3D shape poses as well as joint-space searches), object identification/classification, and platform localisation. Furthermore, the method is shown to be robust in cluttered or non-segmented point cloud data as well as being robust to measurement uncertainty and extrinsic sensor calibration.

## 1. Introduction

The capability for robotic agents to establish beliefs about their environment is fundamental. These beliefs often relate to objects whose geometry is known but their presence and location needs to be established. Three problems typically arise. Given point cloud data: (i) what is the most likely pose of an object of known geometry believed to be present in the data? (ii) which object, from among a set whose geometries are known, is most likely to be present in the data? (iii) where is the agent in a world whose geometry is known? We call these problems, ‘*where is it?*’, ‘*what is it?*’, and ‘*where am I?*’.

The Iterative Closest Point (ICP) algorithm is often used to address these problems. It was introduced by [1] for point cloud matching and is a frequently applied method in object pose estimation [2,3], object classification [4,5] and localisation [6,7]. The ICP method minimises the error between a point cloud and a geometric model [8]. The motivation for minimising error (or other error-based metrics) is that an object, when placed in the correct pose, should reproduce the observed range or point cloud measurements (i.e., zero error). A limitation of ICP is that it is not robust if the point cloud is unsegmented or noise is present.

The method of this paper, which we call Maximum Sum of Evidence, overcomes this limitation by seeking to determine the pose that is most evidenced in the point cloud. The paper builds from [9,10] and demonstrates the efficacy of an evidenced-based approach to addressing ‘*where is it?*’, ‘*what is it?*’, and ‘*where am I?*’ problems.

The paper is organised as follows. Section 2 articulates the limitations of using error-based minimisation. Section 3 details the MSOE algorithm in two parts: (i) Section 3.1 describes an objective function based on the conditional likelihood of range measurements; (ii) Section 3.2 explores searching heuristics that employ the described objective function. The algorithm is demonstrated in Section 4 for its ability to answer ‘*where is it?*’, ‘*what is it?*’, and ‘*where am I?*’ type questions that frequently arise as required perception capabilities in robotics and automation. Section 5 further evaluates the performance of the MSOE estimator in examples that are known to challenge ICP. Concluding remarks are provided in Section 6.

## 2. The Limitations of Using Error-Based Minimisation in Pose Estimation Problems

Error-based minimisation is commonly employed in pose estimation problems where point cloud or range measurements are provided as input [11,12]. This is intuitive as an estimate that reproduces the observed range measurements, Z, will result in zero cost. As such, a search heuristic can be applied that is guided by an objective function that seeks to find a pose hypothesis that minimises an error-based metric. However, the results are prone to error in real data sets.

**Figure 1:**
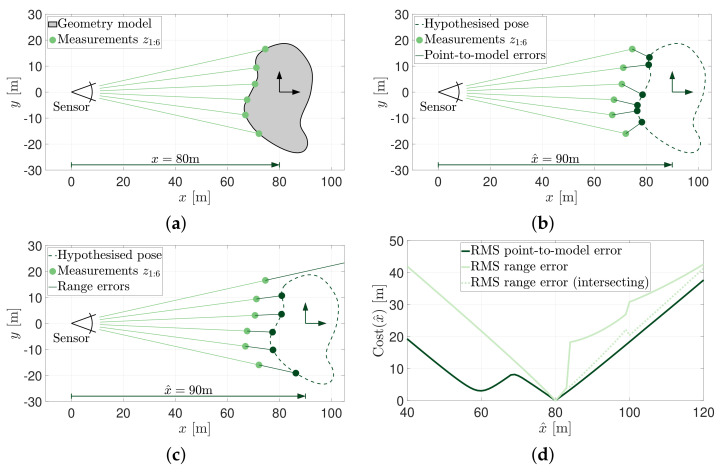
Cost-based metrics and their ability to provide for the estimation of object pose when provided with a perfect measurement set. (**a**) Six range measurements of a geometric body located at x=80 m. (**b**) Point-to-model errors when the geometry model is hypothesised to be located at $\hat{x}=90$ m. (**c**) Range measurement errors when the geometry model is hypothesised to be located at $\hat{x}=90$ m. (**d**) RMS point-to-model and range error cost metrics for hypotheses [40 m $\leq \hat{x}\leq 120$ m].

Consider, for example, the object of known geometry shown in Figure 1a which is free to move in the *x*-coordinate towards or away from the sensor. The geometry, in truth, is located at x=80 m which produces the range measurements, $z_{1:6}$. Two cost-based metrics could readily be applied to this problem to estimate the lateral position of the geometry. The first is illustrated in Figure 1b whereby the point-to-model cost of a measurement is calculated as the Euclidean distance to the closest surface on the geometry model located by the position hypothesis, $\hat{x}$. The overall cost of the hypothesis, cost$\left(\hat{x}\right)$, can be taken as the sum or root-mean-square (RMS) of these errors. The point-to-model errors are shown for a hypothesis of $\hat{x}=90$ m. Figure 1c illustrates the use of range measurement error for the same position hypothesis. Here, the cost is measured as the difference between actual observations and expected observations. Note that the first range measurement, $z_{1}$, no longer hits the geometry in the $\hat{x}=90$ m position, and has its error measured against the maximum range of the sensor.

Equal arguments can be made for either removing or retaining the non-intersecting beam. Retaining the measurement will cause a discontinuity in the cost function as the RMS of range error will rise significantly when beams ‘fall off’ the model. Removing the non-intersecting measurement will misleadingly reduce the cost as a hypothesis with no intersecting beams would have theoretically no cost.

Figure 1d shows the cost of position hypotheses between 40 m to 120 m. The dark and light lines indicate the RMS of point-to-model and range errors, respectively. A dashed light line has also been included to indicate the RMS of range errors when only intersecting beams are considered. This figure highlights the immediate attractiveness of using cost-metrics. The globally minimum cost of both cost-metrics is associated with the correct pose of x=80 m. Both cost metrics, as expected, compute the cost of the true pose to be zero.

At surface level, cost-based metrics are appealing as they appear to provide for the correct pose solution. This paper, however, argues that cost-based metrics are an inherently poor metric for measuring hypothesis correctness. This is most easily demonstrated when measurement uncertainty is introduced to the same example problem.

**Figure 2:**
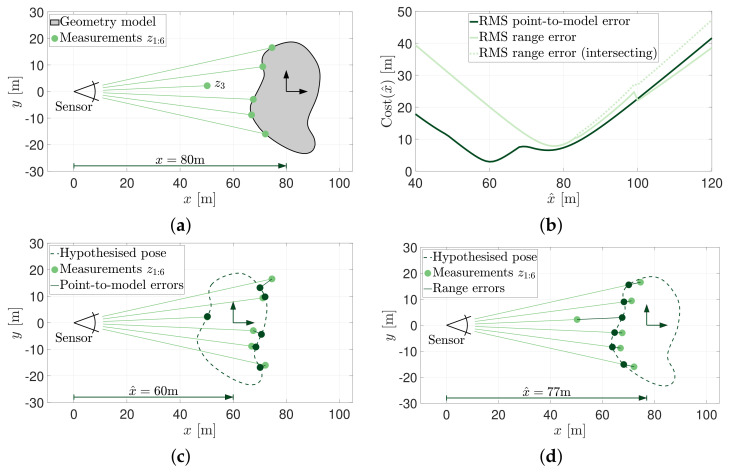
Cost-based metrics and their ability to provide for the estimation of object pose when provided with a corrupted measurement set. (**a**) One of the range measurements, $z_{3}$, is altered to simulate a spurious measurement. (**b**) RMS point-to-model and range error cost metrics when the measurement $z_{3}$ is altered. (**c**) The most likely pose hypothesis using the point-to-model cost metric. (**d**) The most likely pose hypothesis using the range error cost metric.

Figure 2 shows the same pose estimation problem demonstrated in Figure 1, however, a single measurement, $z_{3}$, has been corrupted (Figure 2a). Despite having five perfect measurements of the geometry, this one incorrect measurement is enough to significantly affect the overall cost and change the estimated pose solution. The objective functions when applied to this slightly altered measurement set have been plotted in Figure 2b. Note that there is no longer a zero cost solution as no position could possibly recreate the complete set of observed measurements. More importantly, the globally minimum costs are no longer located at the true solution. The point-to-model error cost metric suggests that the best location for the geometry is at $\hat{x}=60$ m (shown in Figure 2c). Likewise, the range error cost metric suggests that a pose of $\hat{x}=77$ m will result in a globally minimum cost (shown in Figure 2d).

This example demonstrates that cost-based metrics are prone to fail because they are designed to determine where an object must lie in order to recreate the complete measurement set. In real data the assumption that all measurements must belong to the object can not be justified.

Rather than ask ‘*Where does the geometry need lie to recreate the measurements?*’ a better question is ‘*What pose do these measurements support or provide the most evidence towards?*’. This is the fundamental difference in ideology that is adopted by this paper.

## 3. The Maximum Sum of Evidence (MSOE) Method

The Maximum Sum Of Evidence method is predicated on the belief that the most likely pose hypothesis, $H^{★}$, is that which is most supported by the individual range measurement observations. Each of the *N* observed range measurements, $Z=\{z_{1},z_{2},\ldots ,z_{N}\}$, will support a region of the pose hypothesis space, H. We consider a discretised hypothesis space of *M* hypotheses, $H=\{H_{1},H_{2},\ldots ,H_{M}\}$.

The law of total probability relates the marginal probability of the *j*-th pose hypothesis, $P(H_{j})$, to its conditional probability given each range observation, $z_{i}$. It is expressed as,

(1) $$P\left(H_{j}\right)=\sum_{i=1}^{N}P\left(H_{j}|z_{i}\right)P\left(z_{i}\right).$$

The MSOE method seeks to determine the most probable hypothesis, $H^{★}$, i.e., that which maximises the total probability measure. The most evidenced hypothesis is indexed by $j^{★}$ (i.e., $H^{★}=H_{j^{★}}$), which is determined as,

(2) $$j^{★}=\underset{j}{\mathrm{argmax}}\left\{P(H_{j})\right\}=\underset{j}{\mathrm{argmax}}\left\{\sum_{i=1}^{N}P\left(H_{j}|z_{i}\right)P\left(z_{i}\right)\right\}.$$

The conditional probability of a hypothesis given a range observation, $P(H_{j}|z_{i})$, is related to the conditional probability of the hypothesis providing the range observation, $P(z_{i}|H_{j})$ via Bayes’ theorem. It can be expressed as,

(3) $$P\left(H_{j}|z_{i}\right)=\frac{P\left(z_{i}|H_{j}\right)P\left(H_{j}\right)}{P(z_{i})}.$$

Substituting Bayes’ theorem (Equation (3)) into the expression for total probability (Equation (2)) yields,

(4) $$j^{★}=\underset{j}{\mathrm{argmax}}\left\{\sum_{i=1}^{N}\frac{P\left(z_{i}|H_{j}\right)P\left(H_{j}\right)}{P(z_{i})}P\left(z_{i}\right)\right\}=\underset{j}{\mathrm{argmax}}\left\{\sum_{i=1}^{N}P\left(z_{i}|H_{j}\right)P\left(H_{j}\right)\right\}.$$

There are two simplifications that can be made to this equation. The first, is that, in the absence of prior information, all hypotheses can be considered equally likely, i.e.,

(5) $$P(H_{j})=1/M.$$

The second simplification comes from recognising that range measurements have a finite resolution, Δz, and are therefore discrete. The discrete probability of a range measurement can be expressed as,

(6) $$P\left(z_{i}|H_{j}\right)=\int_{z=z_{i}-\Delta z/2}^{z=z_{i}+\Delta z/2}f\left(z_{i}|H_{j}\right)\;dz,$$

which, for small Δz, approximates to,

(7) $$P\left(z_{i}|H_{j}\right)≃f\left(z_{i}|H_{j}\right)\cdot \Delta z.$$

Making use of these two simplifications, Equation (4) becomes

(8) $$j^{★}=\underset{j}{\mathrm{argmax}}\left\{\frac{\Delta z}{M}\sum_{i=1}^{N}f\left(z_{i}|H_{j}\right))\right\}.$$

The scalar terms *M* and Δz do not relocate the maximum and can be removed, leaving,

(9) $$j^{★}=\underset{j}{\mathrm{argmax}}\left\{\sum_{i=1}^{N}f\left(z_{i}|H_{j}\right)\right\}.$$

This result forms the basis of the MSOE algorithm. The most probable hypothesis, $H^{★}$, is that which maximises the sum of conditional measurement likelihoods, $f(z_{i}|H_{j})$.

**Figure 3:**
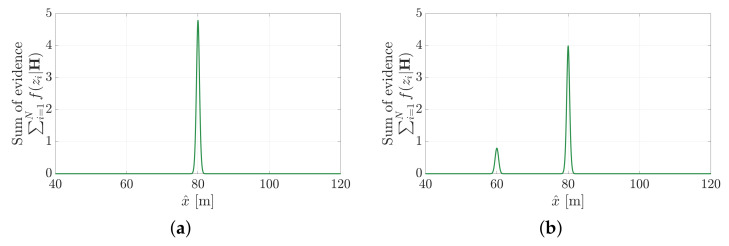
The evidence-based metric suggests that the hypothesis $\hat{x}=80$ m is most likely even when the spurious measurement is introduced. (**a**) Evidence measured across the hypothesis space using the perfect measurement set in Figure 1a. (**b**) Evidence measured across the hypothesis space using the corrupted measurement set in Figure 2a.

Rather than seek to minimise cost of point cloud error, the MSOE method seeks to maximise the conditional likelihood of obtaining the point cloud observations. This provides a significant advantage towards pose estimation in non-segmented point cloud data (which is demonstrated in Section 5.1). Measurements that are not possible under a hypothesis (i.e., $f(z_{i}|H_{j})=0$) will not add support to the hypothesis but will not detract from its likelihood either. Figure 3 shows the evidence metric applied to the measurement sets from the previous section (Figure 1a and Figure 2a). In the first example, all measurements are shown to support the hypothesis that the object is located at $\hat{x}=80$ m. When applied to the second example, the corrupted measurement, $z_{3}$, provides evidence towards the $\hat{x}=60$ m hypothesis, but overall the original measurements still support the true solution.

The two sections that follow provide detail on how to: (i) calculate the conditional range measurement likelihoods, $f(z_{i}|H_{j})$; and (ii) guide searches in the hypothesis space H for the maximum-sum-of-evidence solution, $H^{★}$.

### 3.1. Establishing Conditional Range Measurement Likelihoods, $f(z_{i}|H_{j})$

The conditional measurement likelihood $f(z_{i}|H_{j})$ denotes the conditional likelihood of observing the measurement $z_{i}$ on the *i*-th beam given an assumed object pose $H_{j}$. The calculation of this conditional likelihood requires the use of a measurement model to determine the expected range measurement, denoted ${\hat{z}}_{i|j}$. The LiDAR measurement model (denoted h(·)) is a function that returns the ray-cast range of a beam against the hypothesis-located geometry model. The *j*-th pose hypothesis is modelled by locating the a priori geometry model using $H_{j}$. The range of the *i*-th beam ray-cast against the *j*-th pose hypothesis is expressed as

(10) $${\hat{z}}_{i|j}=h_{i}\left(H_{j}\right).$$

**Figure 4:**
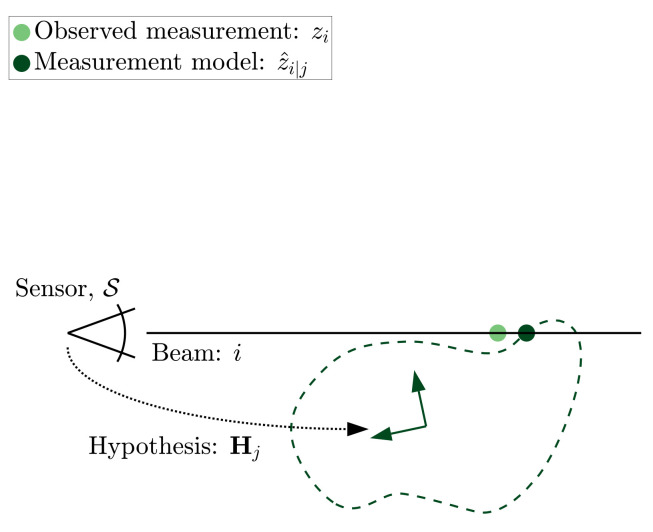
The expected range along the *i*-th beam for hypothesis $H_{j}$ is depicted as ${\hat{z}}_{i|j}$.

Figure 4 illustrates the ray-cast range measurement ${\hat{z}}_{i|j}$ alongside an actual range measurement $z_{i}$. Here, the hypothesis, $H_{j}$, locates the geometry relative to the sensor coordinate frame, *S*.

The support that the observed measurement, $z_{i}$, provides to the hypothesis is determined through the use of a conditional range likelihood model, $f(z_{i}|H_{j})$. The conditional range likelihood model describes where we might expect measurements to occur if the hypothesis were true.

A starting point is to consider the measurement uncertainty of the LiDAR sensor itself. Manufacturers will typically provide the standard deviation of sensor measurement uncertainty, σ.

**Figure 5:**
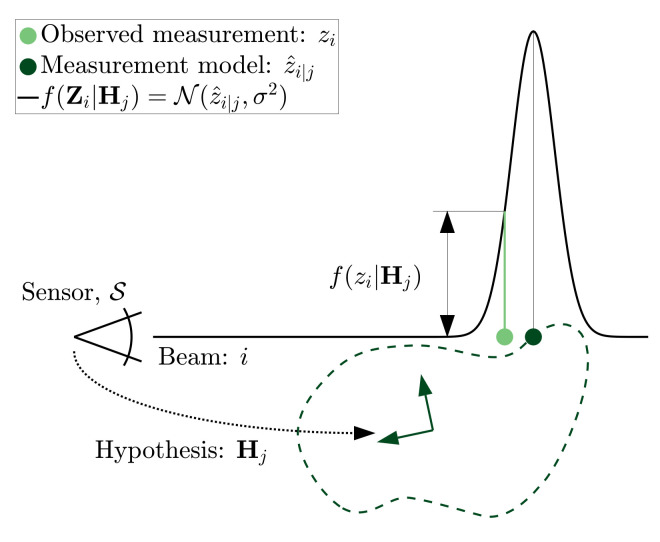
Range likelihood distribution, $f(Z_{i}|H_{j})$, given sensor measurement uncertainty, σ.

Consider Figure 5 which illustrates the range measurement distribution considering only the sensor’s measurement uncertainty. The measurement uncertainty has been exaggerated to σ=3 m for illustrative purposes. In this example, the observed range measurement along this beam, $z_{i}$, is 60 m and is indicated to be a slightly shorter than the range, ${\hat{z}}_{i|j}=64$ m.

The likelihood of observing the actual measurement, $z_{i}$, given the pose hypothesis, $H_{j}$, and the sensor’s measurement uncertainty, σ, is calculated as,

(11) $$f\left(z_{i}|H_{j}\right)=N\left({\hat{z}}_{i|j},\sigma^{2}\right)=\frac{1}{\sqrt{2\pi \sigma^{2}}}\cdot \exp\left(-\frac{{\left(z_{i}-{\hat{z}}_{i,j}\right)}^{2}}{2\sigma^{2}}\right).$$

In this example, a range measurement of 64 m would have provided the most evidence towards hypothesis $H_{j}$, however, the observed range of $z_{i}=60$ m still reasonably evidences the hypothesis.

It is possible to map other sources of uncertainty into the range measurement likelihood function, $f(z_{i}|H_{j})$. By way of example, consider the more general problem of estimating the pose of an object within a greater World coordinate system, *W*, in which the pose of the sensor, $T_{W\to S}$, is also uncertain.

**Figure 6:**
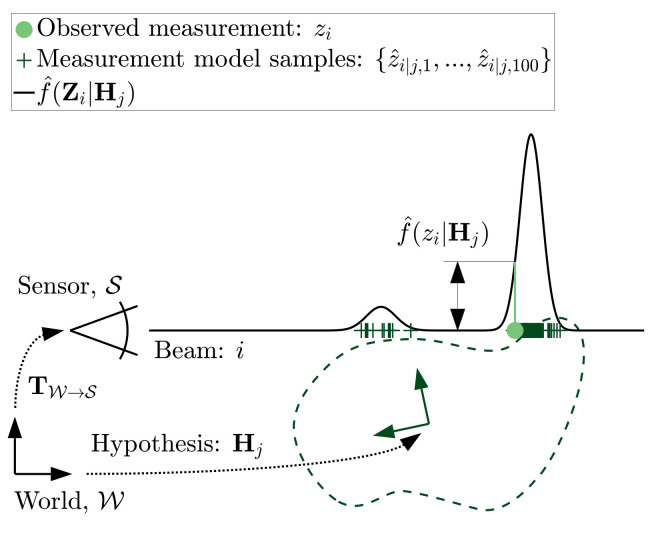
Range likelihood distribution, $\hat{f}\left(Z_{i}|H_{j}\right)$, given sensor measurement uncertainty, σ and sensor pose uncertainty, $\mathrm{Cov}(T_{W\to S})$.

The uncertainty in the sensor’s pose relates to how accurate the extrinsic calibration or registration procedure is. In previous studies we have estimated that this can be determined within several millimetres/milliradians for translational and rotational components, respectively, [13,14]. Figure 6 illustrates the effect of registration uncertainty, $\mathrm{Cov}(T_{W\to S})$, on the range measurement distribution, $f(Z_{i}|H_{j})$.

Sensor pose uncertainty will redirect the beam onto other parts of the geometry model as located by the hypothesis. The redirection of beams can result in abrupt changes to the expected range measurement. The resulting distribution of range measurements now depends on the shape of the geometry. An estimate of the range probability density function, $\hat{f}\left(Z_{i}|H_{j}\right)$, can be established by sampling the range measurement model under perturbed sensor poses drawn from extrinsic calibration uncertainty, w, and adding the sensor’s measurement uncertainty, *v*, to the result. The sampled range measurements along the *i*-th beam under the *j*-th pose hypothesis, ${\hat{Z}}_{i|j}=\left\{{\hat{z}}_{i|j,1},{\hat{z}}_{i|j,2},\ldots ,{\hat{z}}_{i|j,n}\right\}$, are each calculated from

(12) $${\hat{z}}_{i|j,k}=h_{i}\left(H_{j},w_{k}\right)+v_{k},$$

where $w_{k}∼N\left(0,\mathrm{Cov}\left(T_{W\to S}\right)\right)$ and $v_{k}∼N\left(0,\sigma^{2}\right)$.

Figure 6 shows the placement of n=100 sampled range measurements under this new measurement model. The distribution is shown to contain a new mode where the sensor’s pose uncertainty, $\mathrm{Cov}(T_{W\to S})$, could redirect the beam onto a different part of the object’s geometry.

Much work has been done towards the estimation of probability density functions (PDFs) from sampled data, see for example [15]. One such technique is to construct a Gaussian kernel density estimator (KDE) where a small kernel, K(·), is located at each of the ray-cast samples, ${\hat{z}}_{i|j,k}$. The estimated range probability density function, $\hat{f}\left(Z_{i}|H_{j}\right)$, evaluated at $z_{i}$ is then calculated from the summation of kernels

(13) $$\hat{f}\left(z_{i}|H_{j}\right)=\frac{1}{n\cdot h}\sum_{k=1}^{n}K\left(\frac{z_{i}-{\hat{z}}_{i|j,k}}{h}\right),$$

where the Gaussian kernels are described by

(14) $$K\left(x\right)=\frac{1}{\sqrt{2\pi }}\cdot \exp\left(-\frac{x^{2}}{2}\right).$$

The parameter *h* is commonly referred to as the *bandwidth*, and acts as a smoothing parameter between bias and variance. The optimal selection of *h* greatly influences the accuracy to which $\hat{f}\left(\cdot \right)$ represents the true density f(·). Turlach provides a good review of optimal bandwidth techniques in [16]. The performance of $\hat{f}\left(\cdot \right)$ to a selected bandwidth, *h*, can be measured by the integrated squared error (ISE) over the estimated function’s domain

(15) $$\mathrm{ISE}\left(h\right)=\int {\left(\hat{f}\left(Z_{i};h|H_{j}\right)-f\left(Z_{i}|H_{j}\right)\right)}^{2}dZ_{i}.$$

However, this criterion is subject to the sampled range measurement predictions, ${\hat{Z}}_{i|j}$, which are themselves subject to the sampled perturbations, $w_{k}$ and $v_{k}$, used during the raycasting process described in Equation (12). A true assessment of the density estimate’s accuracy is obtained from the expected value of the ISE or *mean integrated squared error* (MISE)

(16) $$\mathrm{MISE}\left(h\right)=E\left[\int {\left(\hat{f}\left(Z_{i};h|H_{j}\right)-f\left(Z_{i}|H_{j}\right)\right)}^{2}dZ_{i}\right].$$

**Figure 7:**
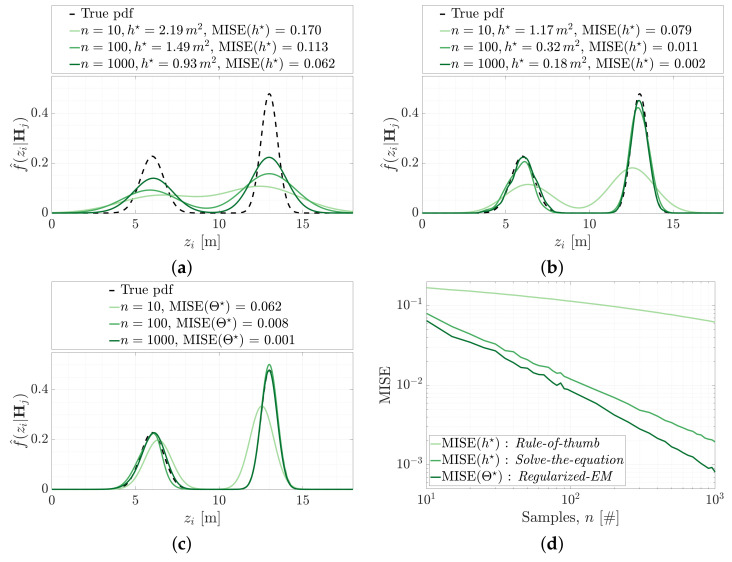
The mean integrated squared error of the estimated conditional range measurement likelihood function, $\hat{f}\left(Z|H_{j}\right)$, as determined using: (**a**) Kernel density estimation using optimal bandwidth, $h^{★}$, established from *Rule-of-thumb* [17]. (**b**) Kernel density estimation using optimal bandwidth, $h^{★}$, established from *Solve-the-equation* [18]. (**c**) Density estimation using optimal parameters, Θ, established from regularised expectation maximisation [19]. (**d**) The MISE for the three estimates are shown for increasing number of samples, *n*.

For Gaussianly distributed sample range measurements, ${\hat{Z}}_{i|j}$, the optimal bandwidth (for minimising MISE(*h*)) can be found from Silverman’s plugin *rule-of-thumb* [17] equation,

(17) $$h^{★}={\left(\frac{4{\hat{\sigma }}^{5}}{3n}\right)}^{1/5},$$

where $\hat{\sigma }$ is the standard deviation of sampled range measurements, ${\hat{Z}}_{i|j}=\left\{{\hat{z}}_{i|j,1},\ldots ,{\hat{z}}_{i|j,n}\right\}$. It is not reasonable to expect the range measurement likelihood $f(Z_{i}|H_{j})$ to be Gaussian, and this bandwidth selection is known to perform poorly on multimodal PDFs (which are shown to arise in Figure 6). Figure 7a demonstrates the performance of kernel density estimation using this optimal bandwidth, $h^{★}$, towards the estimation of a bimodial probability density function $f\left(Z_{i}|H_{j}\right)=0.4\cdot N\left(6,0.7^{2}\right)+0.6\cdot N\left(13,0.5^{2}\right)$. The estimated PDF, $\hat{f}\left(Z_{i};h^{★}|H_{j}\right)$, is shown to be oversmoothed, however, the MISE does decrease as the number of samples *n* increases.

Sheather and Jones present an improved bandwidth selection method that is guided by the sample data [20]. The optimal bandwidth solution, $h^{★}$, seeks to minimise good quality estimates of the MISE. The method is commonly referred to as a *solve-the-equation* approach. Botev et al. improve on this method by removing the assumption that the underlying data is parametric [18]. Removing this assumption prevents the estimator from deteriorating for multimodal densities with widely separated modes. Figure 7b shows the estimated $\hat{f}\left(Z_{i};h^{★}|H_{j}\right)$ using the improved *solve-the-equation* approach of [18].

Expectation maximisation, or EM, provides an alternative way to estimate the probability density function, $f(Z_{i}|H_{j})$. Rather than locating kernels on sample data, EM is an iterative method that seeks to recover the parameters, Θ, describing the underlying modes/species of the sample data, ${\hat{Z}}_{i|j}=\left\{{\hat{z}}_{i|j,1},\ldots ,{\hat{z}}_{i|j,n}\right\}$. This is found by maximising the log-likelihood function describing the likelihood of obtaining each range measurement sample, ${\hat{z}}_{i|j,k}$, given the assumed parameter model, Θ,

(18) $$L\left(\Theta ;{\hat{Z}}_{i|j}\right)=\sum_{k=1}^{n}\log\left(f({\hat{z}}_{i|j,k}|\Theta )\right).$$

The example PDF used in Figure 7 is a Gaussian mixture model constructed from two modes. The first mode provides 40% ($\alpha_{1}$) of the density and is parameterised with $\mu_{1}=6$ m and $\sigma_{1}=0.7\;m^{2}$, the second mode provides the remaining 60% ($\alpha_{2}$) and is parameterised by $\mu_{2}=13$ m, $\sigma_{2}=0.5\;m^{2}$. The parameter space to be estimated is $\Theta =\{\alpha_{1},\mu_{1},\sigma_{1},\alpha_{2},\mu_{2},\sigma_{2}\}$.

A common criticism of EM estimation methods is that the solution must assume a number of underlying modes in the density being estimated and this is usually referred to as the model selection problem. An EM implementation that sought to model the example PDF with three modes would not be able to reduce the over specified model back to two modes. Li et al. [19] provide an EM implementation that can reduce over-specified models by effectively reducing component weightings, α, to zero. The implementation is called the *Regularised EM* algorithm as the likelihood measure (Equation (18)) is regularised by applying a penalty based on the mutual information between sampled *observation data*, ${\hat{Z}}_{i|j}$, and the corresponding *missing data*, Y. Figure 7c shows the estimated PDF using regularised expectation maximisation.

The conditional range measurement likelihood can be estimated using kernel density estimation, $\hat{f}\left(z_{i};h^{★}|H_{j}\right)$, or regularised expectation maximisation, $\hat{f}\left(z_{i};\Theta^{★}|H_{j}\right)$, with both showing low MISE values for reasonably small sample sizes, *n*. Figure 7d shows that the kernel density estimate typically requires 1.5–2.5 as many samples as the regularised expectation maximisation method to achieve the same MISE.

### 3.2. Searching the Hypothesis Space for the Global Maximum Sum of Evidence, $H^{★}$

The sum of evidence measure described in Equation (9) requires the summation of conditional measurement likelihoods. As this paper will show, the evidence metric serves as a very robust objective function for the measure of hypothesis likelihood. A search heuristic is required to determine the hypothesis parameters that yield the maximum sum of evidence. This section explores search heuristic performance using an example 2D search space of a robotic manipulator.

**Figure 8:**
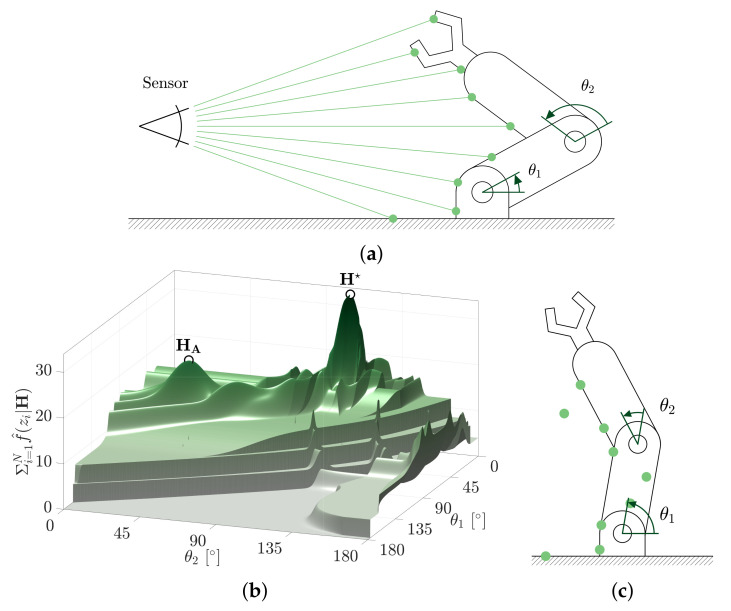
An example 2-DOF problem used to explore the challenges of searching the hypothesis space, H, for the global maximum sum of evidence solution, $H^{★}$. (**a**) Geometry and measurements of the example two degree of freedom estimation problem. (**b**) The sum of conditional likelihoods of the nine range measurements evaluated across the hypothesis space, $H=[\theta_{1},\theta_{2}]$. (**c**) Incorrect local maximum sum of evidence at pose $H_{A}$.

The example robot geometry is parameterised by the hypothesis space H = $\left[0^{\circ }\leq \theta_{1}\leq 180^{\circ },0^{\circ }\leq \theta_{2}\leq 180^{\circ }\right]$ (Figure 8a). Nine range measurements, Z, are obtained from a sensor with the robot located by $H_{truth}=\left[28.6^{\circ },114.6^{\circ }\right]$. Figure 8b shows the evidence measure of Equation (9) mapped across the complete hypothesis space, H. The true hypothesis is shown to produce the global maximum sum of evidence, $H^{★}$, at the correct location, $H_{truth}$. An immediate concern, however, is that many local maximums of evidence exist within this surface. Other robot configurations, such as $H_{A}$ (Figure 8c), are reasonably well evidenced from the range measurements and will result in local maximums.

**Figure 9:**
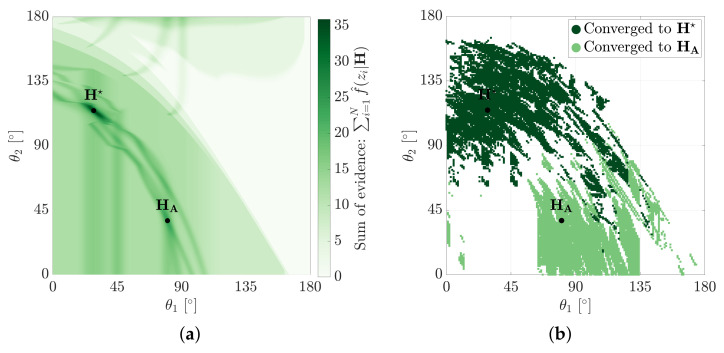
The convergence basin for the MSOE pose, $H^{★}$, and a competing local maximum, $H_{A}$. The basins are determined using a Nelder-Mead solver seeded at every $1^{\circ }$ of $\theta_{1}$ and $\theta_{2}$. (**a**) Evidence measured over the hypothesis space. (**b**) Nelder-Mead seed convergence using the evidence-based metric.

Incorrect pose hypotheses (such as $H_{A}$) can have considerably large basins of convergence. Figure 9b shows the basin of convergence for hypotheses $H^{★}$ and $H_{A}$. The basins were determined by examining the solutions of a Nelder-Mead solver (with side length $10^{\circ }$) seeded in $1^{\circ }$ increments of the hypothesis space, H. Approximately 18% of the seed locations are shown to converge to the MSOE hypothesis, $H^{★}$, however, 11% were also shown to converge to the competing local maximum, $H_{A}$. The remaining 71% of seeds converged elsewhere or not at all.

**Figure 10:**
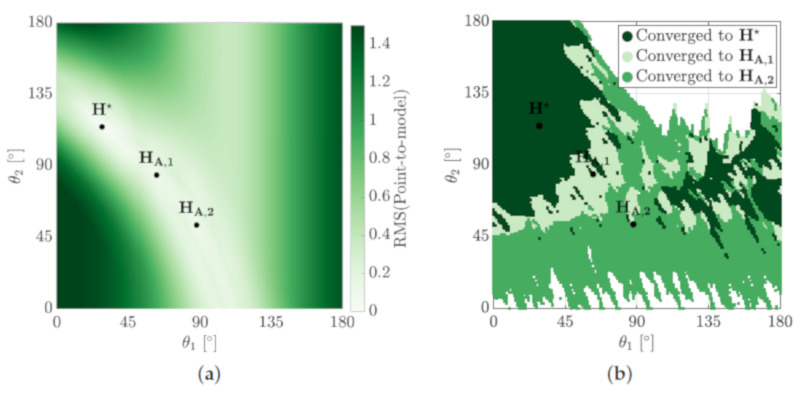
The RMS of point-to-model cost metric has a global minimum at, $H^{★}$, and two competing local minimums at, $H_{A,1}$ and $H_{A,2}$. The basins are determined using a Nelder-Mead solver seeded at every $1^{\circ }$ of $\theta_{1}$ and $\theta_{2}$. (**a**) RMS point-to-model error. (**b**) Nelder-Mead seed convergence on the RMS of point-to-model cost metric.

The local maximums that appear in the evidence-based metric are not dissimilar to the local minimums that arise in cost-based metrics. Figure 10a shows the RMS of point-to-model costs over the same hypothesis space. $H^{★}$ is shown to correctly occur at the global minimum, however, two competing hypotheses $H_{A,1}$ and $H_{A,2}$ are shown to produce relatively large basins of convergence (Figure 10b).

**Figure 11:**
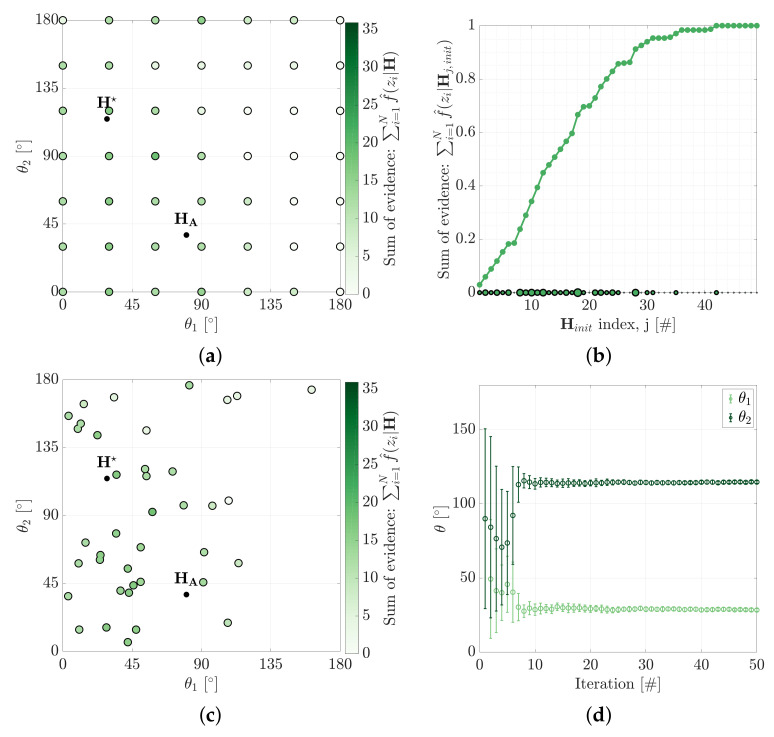
Convergence of the particle search method used to locate $H^{★}$. (**a**) The sum of evidence is determined for an initial set of hypotheses, $H_{init}$. (**b**) A CDF is constructed from this initial measure to guide the concentration of the search for (**c**) the next set of hypotheses, $H_{next}$. (**d**) The solution for this example is shown to converge to $H^{★}$ in approximately 10 iterations of this process, error bars indicate 1 σ of the particle distribution.

The large convergence basins for locally optimal hypotheses promote the need for an alternate searching heuristic that is not seed-dependent. An effective approach is to use a particle-based heuristic to iteratively search the hypothesis space for the MSOE hypothesis, $H^{★}$. The hypothesis space is uniformly sampled over regular intervals, ΔH, to establish an initial hypothesis set, $H_{init}$. Figure 11a shows an initial 49 hypotheses established using $\Delta H=[30^{\circ },30^{\circ }]$.

The next hypothesis set, $H_{next}$, is determined from a cumulative distribution of the evidences determined under $H_{init}$. Uniformly sampling the CDF along its *y*-axis provides a base, $H_{base}$, for where $H_{next}$ should concentrate resampling (Figure 11b). Higher evidenced hypotheses of $H_{init}$ will provide steeper gradient in the CDF which will in turn be resampled more. The circle sizes on the *x*-axis of Figure 11b indicate the number of times that hypothesis is entered into $H_{base}$. The *j*-th hypothesis of the next iteration is determined by adding a small perturbation to $H_{base}$,

(19) $$H_{j,next}∼N\left(H_{j,base},\mathrm{Cov}\left(\Delta H/iteration\right)\right).$$

The covariance, ΔH/iteration, decreases with each iteration, allowing the solution to converge (Figure 11d).

### 3.3. Algorithm Summary

A summary of the MSOE algorithm is provided in Algorithm 1. The algorithm can be implemented by: (i) only considering range measurement uncertainty; but can also be extended to (ii) include the affect of other measurement uncertainties via the sampling method described in Section 3.1.
**Algorithm 1:** Maximum Sum of Evidence Algorithm.
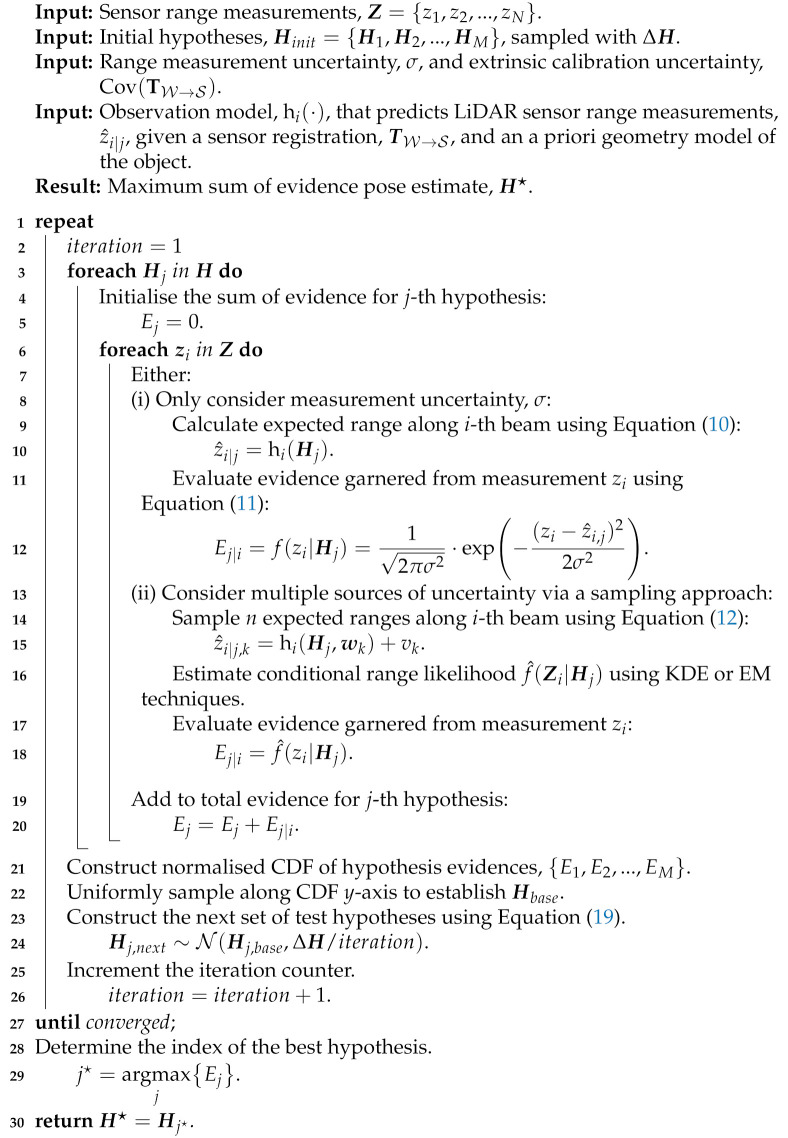



## 4. Sample Problems

The following section evaluates the accuracy of MSOE solutions against a set of candidate problems. The problems are designed to test the algorithm’s capacity to provide three commonly arising perception capabilities: (i) Pose estimation; (ii) Object classification; and (iii) Localisation. These three areas represent ‘*where is it?*’, ‘*what is it?*’, and ‘*where am I?*’ type questions, respectively, which can all be answered via the MSOE algorithm.

### 4.1. Pose Estimation: Answering ‘Where Is It?’-Type Questions

‘*Where is it?*’ questions are a commonly arising question in perception where there is an object whose geometry is known and is expected to appear in the scene. In general, robotic agents need to interact with objects and this requires a belief of where the object is located.

**Figure 12:**
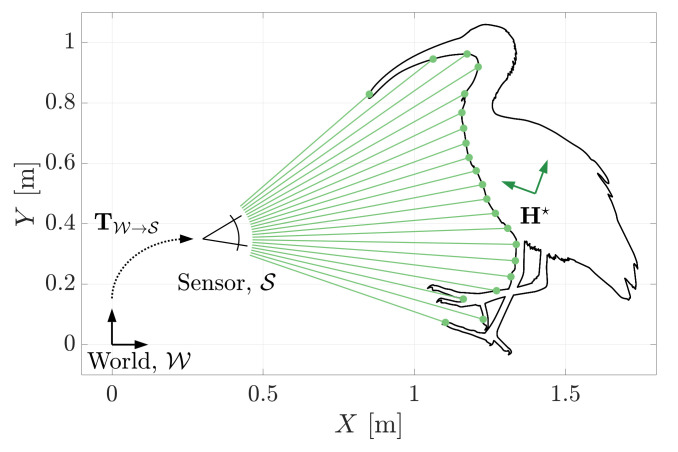
The maximum sum of evidence estimate, $H^{★}$, of a 2D shape’s pose.

Figure 12 demonstrates the evidence-based metric’s ability to estimate the pose of a known 2D geometry. The MSOE pose hypothesis, $H^{★}$, of the 2D ibis shape is shown against the observed 2D range measurements, Z. The shape’s pose is described in three degrees of freedom (DOF). It can rotate ψ about its *Z*-axis after translating *x*, *y* along its *X* and *Y* axes. A measurement set consisting of 21 range measurements was generated from the object at a pose of $H_{truth}=\left[\psi ,x,y\right]=[70^{\circ },1.4\;$m,0.5m].

The pose solution that generated the maximum sum of evidence was determined to be $H^{★}=[70.178^{\circ },1.399\;$m,0.501m]. Comparing $H_{truth}$ and $H^{★}$ directly can be misleading as error in one degree of freedom is often accounted for by error in another. The accuracy of the pose solution is evaluated by determining the maximum geometric displacement of the geometry due to the pose solution error. If points $P_{truth}$ and $P^{★}$ represent the geometry vertices located by $H_{truth}$ and $H^{★}$, respectively, the maximum geometric error, $e_{max}$, is calculated as,

(20) $$e_{max}=\max\left(\left|P_{truth}-P^{★}\right|\right).$$

Under the MSOE solution, the maximum geometric displacement of the 2D shape is $e_{max}=2.9$ mm (achieved using 10 iterations of the search heuristic described in Section 3.2). At 20 iterations, the maximum displacement is reduced to $e_{max}=1.3$ mm and, with the perfect measurement set provided, will, by design, converge to 0 mm.

**Figure 13:**
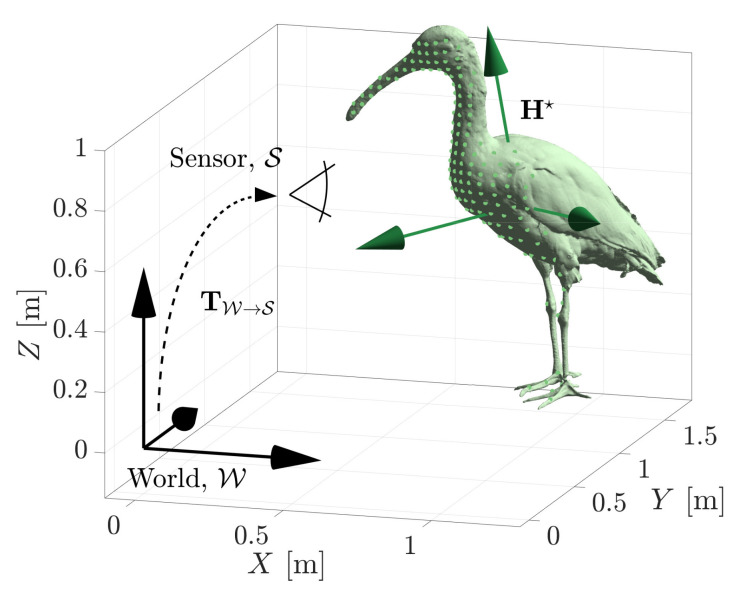
The maximum sum of evidence estimate, $H^{★}$, of a 3D shape’s pose.

The pose estimation problem can be extended to three-dimensional geometries. Figure 13 shows the MSOE pose solution, $H^{★}$, of a 3D ibis geometry estimated against 3D range data. The object’s pose is described in 6-DOF, it can rotate θ, ϕ, ψ and translate *x*, *y*, *z* about and along its *X*, *Y*, and *Z* axes. A measurement set consisting of 150 range measurements was generated from the object at a pose of $H_{truth}=\left[\theta ,\phi ,\psi ,x,y,z\right]=[6^{\circ },8^{\circ },224^{\circ },0.9\;$m,1.2m,0.6m]. Ten iterations of searching the hypothesis space yielded a MSOE pose solution of $H^{★}=[6.48^{\circ },9.95^{\circ },224.23^{\circ },0.900\;$m,1.203m,0.605m], which produces a maximum geometric displacement of $e_{max}=27.4$ mm. Again, this error will continue to decrease with more iterations.

**Figure 14:**
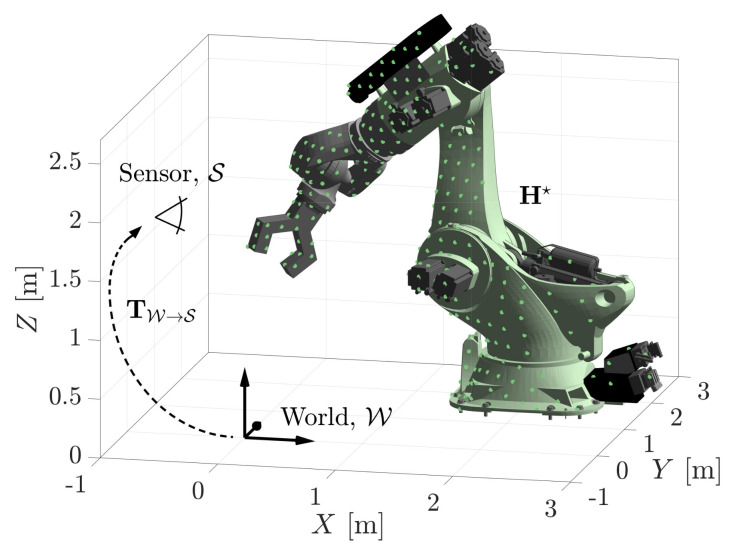
The maximum sum of evidence estimate, $H^{★}$, of a 6-DOF robot’s configuration.

The region of space occupied by an object does not necessarily have to be parameterised by rigid body transformations. Consider the serial linkage robot shown in Figure 14 which is parameterised by its six rotational degrees of freedom, $\theta_{1:6}$. The MSOE algorithm does not require adaptation to joint-space estimation problems. Conditional range measurements likelihoods, $f(Z|H_{j})$, are still calculated as per Section 3.1. The *j*-th hypothesis $H_{j}$ is modelled by articulating the individual linkages, rather than applying a rigid frame transformation to the geometry.

A measurement set of 261 range measurements was generated with the robot configured at $H_{truth}=\left[\theta_{1:6}\right]=\left[10^{\circ },-80^{\circ },120^{\circ },-50^{\circ },30^{\circ },-10^{\circ }\right]$. After 10 iterations of searching, the estimated solution $H^{★}=\left[10.108^{\circ },-79.875^{\circ },119.620^{\circ },-52.160^{\circ },31.122^{\circ },-2.615^{\circ }\right]$ is accurate enough to locate the robot geometry within $e_{max}=44.2$ mm of its true position. The joint-space parameters, representing the angular rotations of the serial linkages, are typically increasing in error from $\theta_{1}$ to $\theta_{6}$. This is because the estimate of each joint parameter relies on the accuracy of the preceding joints, e.g., $\theta_{3}$ cannot be calculated until a sufficiently accurate $\theta_{1}$ and $\theta_{2}$ have been obtained. A further 10 iterations of searching reduces the maximum displacement to $e_{max}=26.2$ mm.

The MSOE estimator can be applied any parameterisable configuration of geometry that affect range measurements, e.g., object scale.

### 4.2. Object Classification: Answering ‘What Is It?’-Type Questions

Answering ‘*What is it?*’ questions is a commonly required capability that arises in perception-related problems. The sum of conditional measurement likelihoods provided by MSOE is a useful classifier for determining which object is present in a point cloud. The evidence-based metric can be used to identify which shape (from a bank of shape hypotheses) would be least surprising to reproduce a set of observed range measurements. This section demonstrates MSOE for its ability to distinguish between different shapes/geometries in point cloud data.

**Figure 15:**
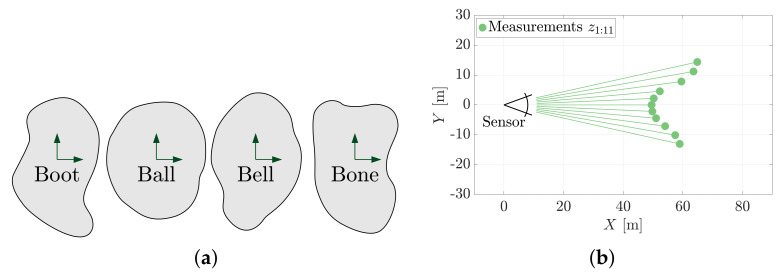
This problem seeks to establish which of four nugget geometries is most likely represented by the range measurements Z. (**a**) Four chicken nugget shapes. (**b**) A measurement set, $Z=\{z_{1:11}\}$, is produced from the *Bone* nugget.

Figure 15 introduces an example object identification problem in which one of four shapes must be determined from a 2D range measurement set. The *Boot*, *Ball*, *Bell* and *Bone* shapes represent the set of possible shapes for chicken nuggets [21]. The MSOE method can be used to robustly distinguish between them or any other set of geometries where such capacity is needed.

**Figure 16:**
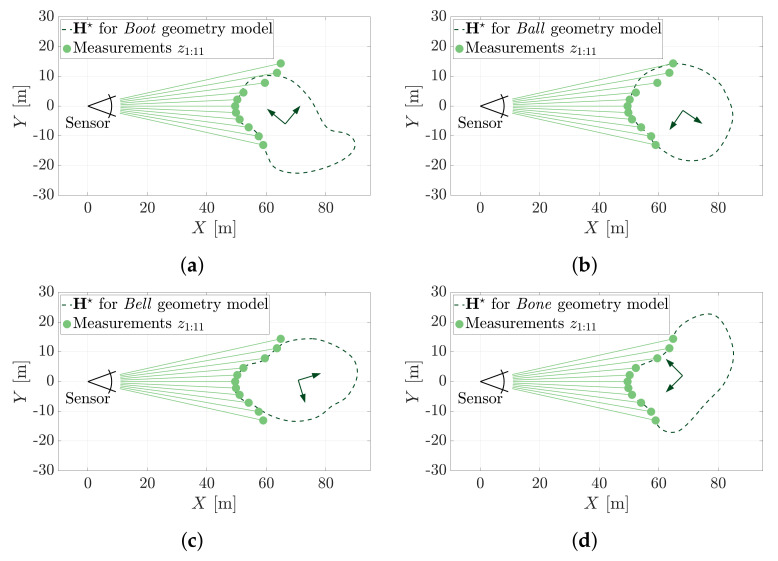
Each geometry is fit to the measurement set using the MSOE algorithm. The maximum sum of evidence measured in these four poses is used to determine which geometry is present in the data. (**a**) The maximum sum of evidence pose of the *Boot* geometry model. (**b**) The maximum sum of evidence pose of the *Ball* geometry model. (**c**) The maximum sum of evidence pose of the *Bell* geometry model. (**d**) The maximum sum of evidence pose of the *Bone* geometry model.

Figure 16 shows the pose hypotheses, $H^{★}$, that yield the maximum sum of evidence for each nugget geometry model when fitted to the measurement set shown in Figure 15b.

**Figure 17:**
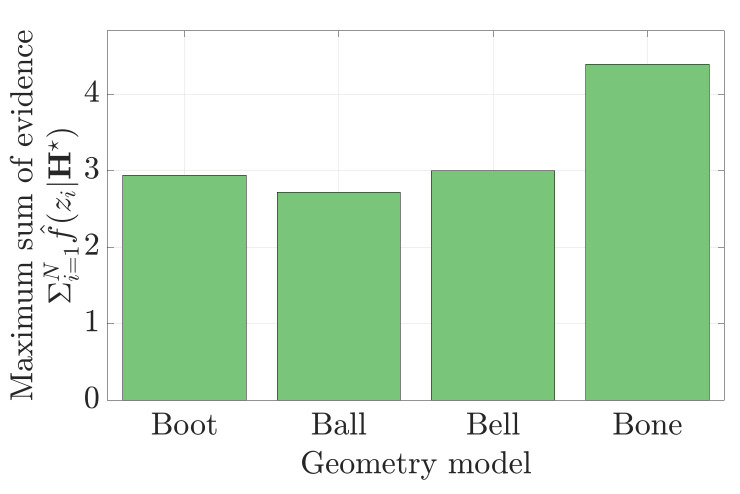
The maximum sum of evidence for the estimated pose of each geometry model. note that the correct geometry, the *Bone*, is most evidenced by the point cloud data.

Figure 17 shows the maximum sum of evidence determined for each of the four nugget shapes. The example shows that the *Bone* nugget was most evidenced by the 11 range measurements. In this example, MSOE is demonstrated to simultaneously answer the questions of ‘*where is it?*’ and ‘*what is it?*’ The discriminating power of the evidence-based metric towards object identification would be much higher if the pose was already known (i.e., just a ‘*what is it?*’ question).

**Figure 18:**
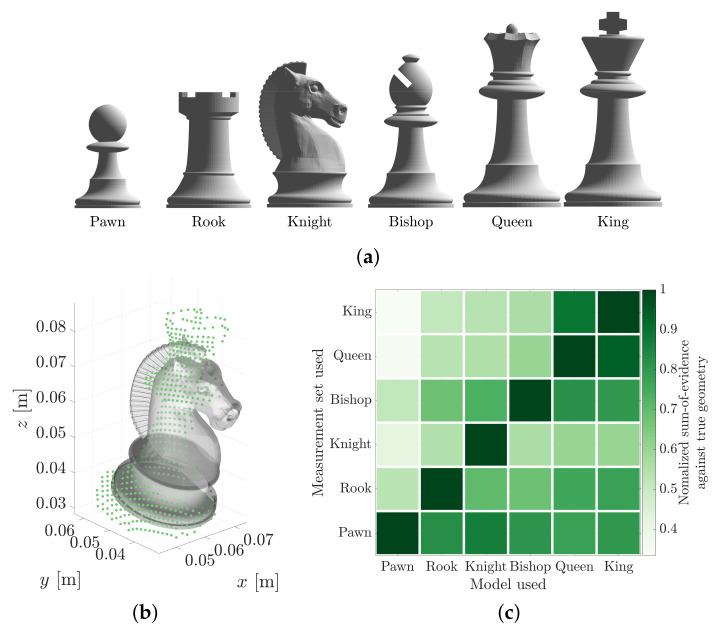
The maximum sum of evidence of a pose estimate can be used to identify which 3D geometry is most capable of reproducing the observed measurements. (**a**) Six chess piece geometry models. (**b**) The maximum sum of evidence pose ($H^{★}$) of the *Knight* geometry as estimated against point cloud measurements of the *Queen*. (**c**) The maximum sum of evidence obtained from each geometry model normalised against the maximum sum of evidence obtained using the true geometry model.

The object identification problem can be extended to three-dimensional shapes. Figure 18 explores the problem of classifying 3D chess piece geometries in 3D point cloud data. Six point clouds were established using each geometry model. The MSOE algorithm was used to determine a 6-DOF pose estimate, $H^{★}$, for each geometry model within each of the six point clouds. Figure 18b shows the estimated pose of the *Knight* geometry model within the point cloud established from the *Queen* geometry model. In this case, the MSOE measured with the *Knight* geometry model was only 55% as high as the MSOE measured with the true geometry model, the *Queen*. Figure 18c plots a confusion matrix normalising each sum-of-evidence score against that obtained with the true geometry model.

The results show that, in all six cases, the correct geometry produces the highest sum of evidence. The determination of the *King* and *Queen* pieces proved to be the most difficult to differentiate from each other. The two geometries are very similar: it is possible to recreate the *Queen* measurement set from the *King* and *vise versa* (approximately 92% in each case). Not all model/measurement tests shared this symmetry. The *Pawn* geometry model could only produce 33% of the *King* point cloud MSOE, whereas the *King* geometry could obtain 79% of the *Pawn* point cloud’s MSOE. This asymmetry occurs because the *pawn* is much smaller than the *king* and can only reproduce a fraction of the measurements.

### 4.3. Localisation: Answering ‘Where Am I?’-Type Questions

The ICP algorithm is frequently applied in Simultaneous Localisation and Mapping (SLAM) to estimate the incremental scan-to-scan frame transformation [22,23]. We demonstrate the MSOE algorithm’s capability towards localisation of an already established map, however, this map could be replaced with that of a previous scan. In this way, we are effectively inverting the ‘*where am I?*’ problem into a ‘*where is it?*’ problem.

**Figure 19:**
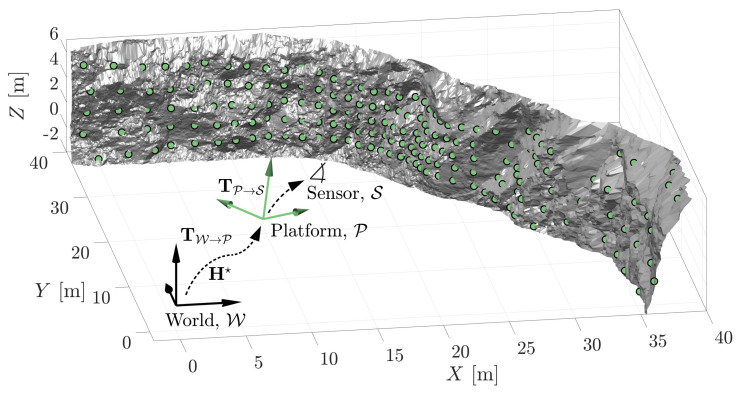
Example localisation problem. The MSOE algorithm is demonstrated to provide the pose of the platform frame, *P*, with respect to the World coordinate frame, *W*.

Given an a priori map of the immediate terrain described in the world frame, *W*, the MSOE algorithm can be used to locate the pose of the platform frame, *P*. Rather than locate a geometry model, the *j*-th hypothesis, $H_{j}$, locates the frame of the platform. The MSOE pose estimate, $H_{j}$, is the platform pose most likely to provide the range measurements, Z. In this way we are estimating the transform $T_{W\to P}$ rather than $T_{W\to Model}$. Figure 19 shows an estimated platform pose calculated from measurements of a 3D terrain model.

A measurement set, Z, of 185 range measurements was established from a true platform pose, $H_{truth}=[4^{\circ },6^{\circ },37^{\circ },9$ m, 15 m, 1 m] and a fixed platform to sensor pose, $T_{P\to S}$. The MSOE estimate for the world-to-platform transformation, $T_{W\to P}$, is $H^{★}=[4.02^{\circ },5.95^{\circ },$$37.00^{\circ },8.997$ m, 15.004 m ,0.982 m]. The origin of the estimated coordinate frame has a Euclidean translational and rotational error of 18.2 mm and $0.054^{\circ }$, respectively. Most of the error is located within the *z*-coordinate of the estimated platform pose. This is predictable as the terrain does not include a floor, and provides very little geometric information with which to estimate the height of the platform.

## 5. What Challenges Does MSOE Overcome?

The following sections are provided to give insight into the performance of the MSOE algorithm when dealing with commonly arising challenges in pose estimation. The behaviour of the evidence-based MSOE algorithm is compared to that of the cost-based Iterative Closest Point (ICP) method. ICP is considered to be the state of the art method for object pose estimation in point cloud data.

### 5.1. MSOE Does Not Require Point Cloud Segmentation

The estimation of object pose in cluttered point clouds presents a significant challenge to algorithms that employ cost-based metrics. While segmentation routines exist, challenging environments can produce unwanted, and uncontrollable, range measurements, e.g., environments where dust is present [9].

Methods such as the Iterative Closest Point method seek to minimise the distance between the point cloud measurements and the geometry model. The problem with this approach is that it is based in the assumption that all points within the point cloud belong to the model. ICP yields excellent results when this is true, but poor results otherwise.

**Figure 20:**
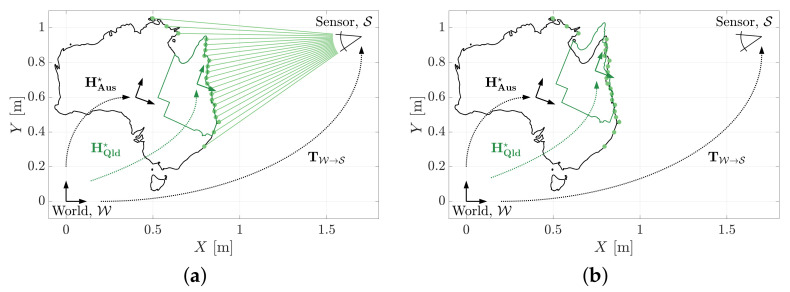
Models of Australia (black) and Queensland (green) fit to a point cloud measurement set. (**a**) The MSOE algorithm is able to locate both geometries, however (**b**) the ICP algorithm is unable to locate the Queensland model without prior segmentation of the point cloud.

Consider the estimation problem illustrated in Figure 20. A point cloud has been obtained from a two dimensional shape representing the Australian mainland. Figure 20a shows the MSOE pose of both an Australian border ($H_{Aus}^{★}$) and Queensland border ($H_{Qld}^{★}$) estimated from the complete measurement set.

Figure 20b shows the results of this example repeated with the ICP method. The border model of Australia is correctly located, however, the estimated pose of the Queensland border is shown to be in considerable error. The failure mechanism here is similar to that observed in Section 2. The measurements that do not belong to Queensland produce an undesirable influence on the ICP pose solution. MSOE effectively ignores measurements that do not add evidence.

**Figure 21:**
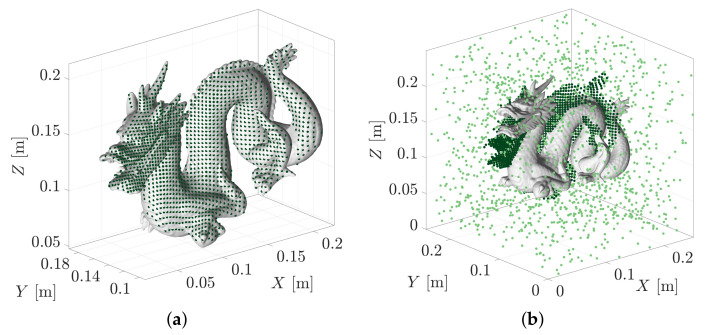
The ICP point-to-plane fit is unable to determine the pose of the model in the cluttered point cloud. (**a**) ICP point-to-plane fit of Stanford Dragon model to segmented point cloud. (**b**) ICP point-to-plane fit of Stanford Dragon model to the same point cloud with an equal number of uniformly random measurements (i.e., 50% clutter).

Figure 21a shows the ICP fit of the Stanford Dragon geometry model to a simulated LiDAR point cloud of 1554 range measurements. The pose estimate was obtained using the base ICP implementation provided by Point Cloud Library [24]. Figure 21b shows how the pose solution is corrupted when an additional 1554 measurements of uniformly distributed clutter/noise are introduced. The failure that occurs here is a result of the cost-based metric treating each point as equally important in minimising the point-to-model distances.

**Figure 22:**
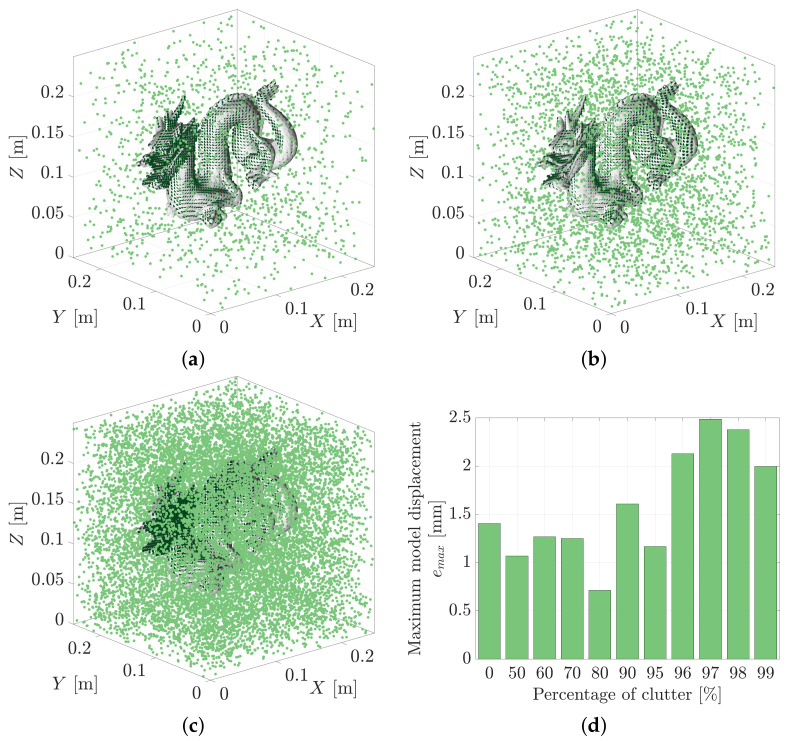
The maximum sum of evidence solution, $H^{★}$, is plotted against the (**a**) 50%, (**b**) 70%, and (**c**) 90% clutter measurement sets. Further testing was conducted on measurement sets consisting of up to 99% clutter (153,846 clutter: 1554 true). (**d**) The maximum geometric displacement of the dragon models, $e_{max}$, was found to reach up to 2.5 mm in the 97% clutter test.

The evidence-based metric used by MSOE is guided by the conditional likelihoods of obtaining each measurement, $z_{i}$, under an assumed pose hypothesis, $H_{j}$. Spurious measurements may be likely under some of the tested hypotheses, however, they will not coherently support the same pose hypothesis in the way that true measurements support the correct pose. From an information perspective, they do not detract from the available information contained within the true measurement set, they simply do not provide any additional information. Figure 22 shows the MSOE pose solutions for measurement sets consisting of 50% to 90% clutter. The point clouds in this figure contain the original 1554 correct measurements and then add in an additional 1554, 3626 and 13,986 clutter measurements for 50%, 70% and 90% clutter experiments, respectively. The MSOE pose estimate is shown to remain robust to cluttered point clouds even up to the 99% clutter test which contains 153,846 clutter measurements.

The largest observed model displacement, $e_{max}$, was 2.5 mm and was found to occur in the 97% noise test. Interestingly, the most accurate pose was found in the 80% noise test which produced a solution that displaced the model by 0.7 mm (Figure 22d). This suggests that the algorithm is operating robustly when random clutter is introduced to the point cloud.

### 5.2. MSOE Is Robust to Range Measurement Uncertainty, σ

Range sensors do not provide perfect range measurements as simulated in the previous sections. Section 3.1 provided discussion on how sensor measurement uncertainty affects the range distribution (as was illustrated in Figure 6). Range sensor measurement accuracy is typically reported as having zero bias and $\sigma^{2}$ variance. For example, the Velodyne VLP-16 sensor is rated to σ = 30 mm [25], whereas survey-grade LiDAR such as the FaroFocus$3^{D}$ is rated to a more accurate σ = 0.3 mm [26].

**Figure 23:**
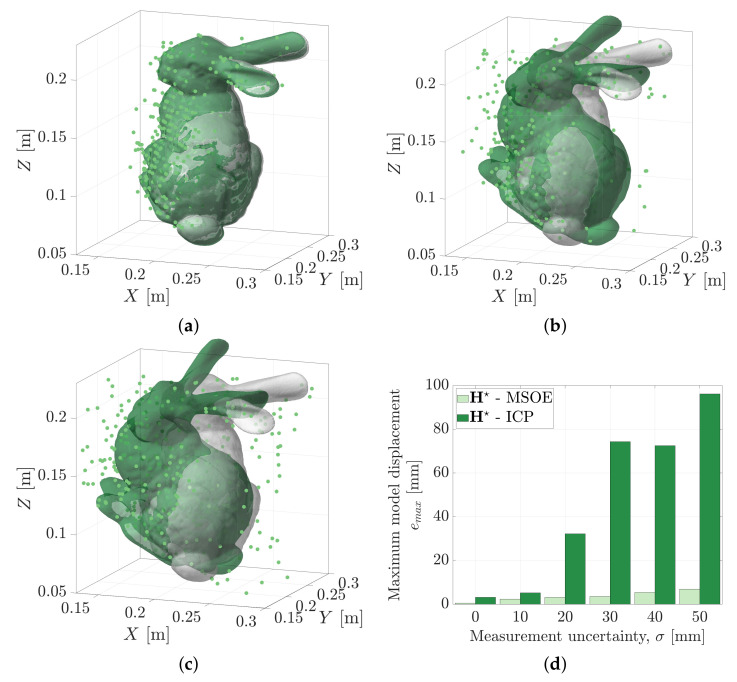
ICP (green) and MSOE (grey) pose solutions obtained for simulated measurement uncertainties of: (**a**) σ=10 mm; (**b**) σ=30 mm; (**c**) σ=50 mm. (**d**) Maximum geometric displacement for increasing measurement uncertainty, σ.

Cost-based metrics such as ICP do not lend themselves to handling measurement uncertainty, whereas MSOE is based in the range measurement space. Figure 23 shows ICP pose estimates (green) of the Stanford Bunny geometry. The solutions are estimated against range data with increasing measurement uncertainty, σ = 10 mm to 50 mm. The pose determined from the Iterative Closest Point method is shown to rotate the geometry, aligning its major-axis with the direction of range measurements. This behaviour occurs as it is the best way to minimise the point-to-model error metric driving the solution.

Maximum sum of evidence pose estimates, $H^{★}$, are plotted in grey alongside the ICP solutions. There is no visible difference in the MSOE pose estimates as measurement uncertainty is increased. The maximum geometric displacement, $e_{max}$, of the bunny geometry model is indicated for both ICP and MSOE in Figure 23d. Under the cost-based ICP approach, the maximum geometric displacement is shown to increase as high as 96.1 mm. When using the MSOE approach, the geometry model is shown to displace up to 6.9 mm (which occurred in the σ = 50 mm test). The range measurement error used to construct this test is sampled from an unbiased Gaussian, $N(0,\sigma^{2})$. Geometric displacement of the geometry using the MSOE solutions is most likely due to any bias that occurred in the as-sampled range errors.

## 6. Conclusions and Significance

This paper applies a single evidence-based method towards the problems of pose estimation, object classification and platform localisation. The solutions to these three problem categories are shown to be accurate when assessed under the metric of maximum geometric displacement.

Poses are obtained for two dimensional and three dimensional objects and the method is shown to extend to finding solutions in other hypothesis spaces, e.g., robot joint space. The maximum sum of evidence measure is shown to provide a good classifier of objects from point cloud data. An object is better suited to a point cloud if it contains geometry that is more likely to reproduce the range measurements. Finally, MSOE is shown to provide for accurate platform localisation against a priori map geometries.

The main contribution of the MSOE approach lies in its robustness to segmentation and measurement uncertainty. This robustness is achieved by removing the need for an assumed point cloud correspondence. The algorithm’s performance is tested on unsegmented data and compared to the results obtained with ICP (a cost-based method). Pose estimates are shown to be robust to unsegmented measurements of a scene as well as unsegmented random noise. MSOE is also demonstrated to find accurate pose estimates in point cloud data produced with significant range uncertainty.

The final conclusion of this paper is that the MSOE algorithm does not require any variation to handle the challenges associated with pose estimation in cluttered point clouds, e.g., segmentation. The algorithm takes unprocessed range measurements as input and provides pose estimates, object classification, or localisation as output. There are no tuning parameters required (e.g., nearest neighbour regions or voxel size). It merely requires an assumed geometry model and a representative magnitude of measurement or extrinsic calibration uncertainty in order to be configured.

We have used the MSOE algorithm in our work in Mining equipment automation. The benefits of the algorithm far outweigh the increased computational cost and we recommend its use to others who are seeking to solve ‘*where is it*?, ‘*what is it*?’, and ‘*where am I*?’ problems.

## Abbreviations

The following abbreviations are used in this manuscript:

MSOE	Maximum Sum Of Evidence
ICP	Iterative Closest Point
MISE	Mean Integrated Square Error
ISE	Integrated Square Error
KDE	Kernel Density Estimation
EM	Expectation Maximisation
LiDAR	Light Detection Furthermore, Ranging
DOF	Degree Of Freedom
PDF	Probability Density Function
CDF	Cumulative Density Function
